# Editorial

**Published:** 1840-12-19

**Authors:** 


### THE MEDICAL EXAMINER.

PHILADELPHIA, DECEMBER 19, 1840.

### AUTHORS AND REVIEWERS.

It was a resolution of Hume's, which he inflexibly maintained, not to reply to any reviewer. Never author had stronger provocation to enter the field in his own defence. After the publication of the first volumes of his history of England, he was assailed, as he tells us, by one cry of reproach, disapprobation, and even detestation. English, Scotch, and Irish, Whig and Tory, Churchman and Sectary, Freethinker and Religionist, Patriot and Courtier, united in their rage against him in an universal clamour. He calmly wrote on, confident in himself, and relying on the "sober second-thoughts" of the public, which did not disappoint him. This philosophy, or callousness of feeling, seems to be a desideratum with some medical writers of the day. So voracious has constant feeding made the appetite for praise, that any review short of an unqualified puff, is now almost sure to elicit a personal squabble with the angry author or some zealous friend. It has been our good fortune, in our own brief career, not to encounter the hostility of any of the genus irritabile. Content with bestowing commendation where we have thought it deserved, we have, in general, preferred to leave what is faulty to the obscurity to which it usually sinks. Without shrinking from a full and free discussion of vexed questions, it has been our constant aim to pay a just attention to the proverbially irritable feelings of authors.

We regret to see that the occasional acerbity of reviewers is sometimes met by very undue ferocity on the part of authors. For a single stone cast, a whole volley is thrown back. Sneers are retaliated by personalities, and a contest ensues degrading to the dignity of science. We cannot help thinking that the bad taste of a snarling critique is exceeded by the folly of replying to it. A really valuable work is safe before the tribunal of public judgment. The malevolence of critics reacts upon themselves, and their misrepresentations may in general be left to the correcting influence of time. Homer lives, but Zoilus is forgotten.

